# Cyberincivility in the Massive Open Online Course Learning Environment: Data-Mining Study

**DOI:** 10.2196/12152

**Published:** 2018-12-21

**Authors:** Jennie C De Gagne, Kim Manturuk, Hyeyoung K Park, Jamie L Conklin, Noelle Wyman Roth, Benjamin E Hook, Joanne M Kulka

**Affiliations:** 1 School of Nursing Duke University Durham, NC United States; 2 Learning Innovation Duke University Durham, NC United States; 3 Health Sciences Library University of North Carolina, Chapel Hill Chapel Hill, NC United States; 4 Social Sciences Research Institute Duke University Durham, NC United States; 5 School of Medicine University of South Carolina, Greensville, SC United States; 6 Nursing Sandhills Community College Pinehurst, NC United States

**Keywords:** aggression, cyber incivility, discussion forums, education, massive open online courses

## Abstract

**Background:**

Cyberincivility is a pervasive issue that demands upfront thinking and can negatively impact one’s personal, professional, social, and educational well-being. Although massive open online courses (MOOCs) environments could be vulnerable to undesirable acts of incivility among students, no study has explored the phenomena of cyberincivility in this learning environment, particularly in a health-related course in which mostly current or eventual health professions students enroll.

**Objective:**

This study aimed to analyze the characteristics of text entries posted by students enrolled in a medicine and health care MOOC. The objectives were to (1) examine the prevalence of posts deemed disrespectful, insensitive or disruptive, and inconducive to learning; (2) describe the patterns and types of uncivil posts; and (3) highlight aspects that could be useful for MOOC designers and educators to build a culture of cybercivility in the MOOC environment.

**Methods:**

We obtained data from postings in the discussion forums from the MOOC Medical Neuroscience created by a large private university in the southeast region of the United States. After cleaning the dataset, 8705 posts were analyzed, which contained (1) 667 questions that received no responses; (2) 756 questions that received at least one answer; (3) 6921 responses that applied to 756 posts; and (4) 361 responses where the initiating post was unknown. An iterative process of coding, discussion, and revision was conducted to develop a series of a priori codes. Data management and analysis were performed with NVivo 12.

**Results:**

Overall, 19 a priori codes were retained from 25 initially developed, and 3 themes emerged from the data—Annoyance, Disruption, and Aggression. Of 8705 posts included in the analysis, 7333 (84.24%) were considered as the absence of uncivil posts and 1043 (11.98%) as the presence of uncivil posts, while 329 (3.78%) were uncodable. Of 1043 uncivil posts analyzed, 466 were coded to >1 a priori codes, which resulted in 1509 instances. Of those 1509 instances, 826 (54.74%) fell into “annoyance”, 648 (42.94%) into “disruption”, and 35 (2.32%) into “aggression”. Of 466 posts that related to >1 a priori codes, 380 were attributed to 2 or 3 themes. Of those 380 posts, 352 (92.6%) overlapped both “annoyance” and “disruption,” 13 (3.4%) overlapped both “disruption” and “aggression,” and 9 (2.4%) overlapped “annoyance” and “aggression,” while 6 (1.6%) intersected all 3 themes.

**Conclusions:**

This study reports on the phenomena of cyberincivility in health-related MOOCs toward the education of future health care professionals. Despite the general view that discussion forums are a staple of the MOOC delivery system, students cite discussion forums as a source of frustration for their potential to contain uncivil posts. Therefore, MOOC developers and instructors should consider ways to maintain a civil discourse within discussion forums.

## Introduction

### Background

Cyberincivility—defined as disrespectful, insensitive, or disruptive internet behavior—is a pervasive problem. This widespread misbehavior can negatively affect personal, professional, and social well-being [1]. With the proliferation of online classes, more and more health professions students are exposed to these behaviors, which include negative comments about patients, peers, the work environment, or the profession itself; profanity, breaches of patient confidentiality, and discriminatory language can be found on social media sites, blogs, and even in Web-based discussion forums [1,2].

The problem is growing as an increasing number of learners are taking online courses to obtain their degrees and seek out distance-based learning for their personal and professional growth [3]. Despite the popularity of such courses and their potential to address geographical and financial barriers, the Web-based format can leave participants in the anonymity, asynchronicity, and casual instant discourse that characterizes cyberspaces [4]. As a result, discussions may be less effective than they would be if participants were face-to-face in a traditional classroom.

While participants have less anonymity and more personal relationships in a traditional closed online course in higher education, it is not the case when classes are open to the public and mostly free of charge as in massive open online courses (MOOCs) [5]. As such, MOOCs, by virtue of a large number of students and their anonymity, leave participants particularly susceptible and vulnerable to undesirable acts of incivility. However, no study has explored forms and patterns of cyberincivility in the MOOC environment, particularly in a health professions course. Thus, this study investigates this issue and discusses its implications for health professions education.

### Pedagogy of Massive Open Online Courses

Although MOOCs have been available since 2008, their popularity blossomed in 2012 when Stanford University and Michigan Institute of Technology offered a joint course in artificial intelligence; some 160,000 individuals from around the world registered for the class [6,7]. Other disciplines including health professions rapidly followed suit. For example, Goldschmidt and Greene-Ryan created a mini MOOC titled “*Gateway to Online Learning* ” aimed at registered nurses who had been in the field and out of the classroom for a long period [8]. They described their course as a tool to prepare nursing students for the rigors of Web-based learning and viewed MOOCs as a means of allowing more health profession students better access to higher education [8,9]. Liyanagunawardena et al agreed, calling *MOOCs an* effective strategy for gaining knowledge in the health care field [10]*.* However, MOOCs are not without controversy.

MOOCs differ from traditional and even online college classes in several key ways. First, learners who enroll are generally not required to complete prerequisite courses or demonstrate course readiness [5]. Second, course content is usually delivered through short videos, and there is little, if any, direct student-to-professor interaction [5,11]. This means that MOOCs rely more on peer-to-peer discussions to resolve questions and problems than a traditional course where an instructor would answer most questions [5]. Finally, MOOCs are more likely than traditional courses to be taken as stand-alone experiences [5]. Students in a MOOC may not have taken or do not plan to take other courses. They may not have the same writing or study skills that many college students have. This means that they may rely, to a greater extent, on class discussion forums for help with their coursework [12]. Thus, discussion forums play a key role in the MOOC environment, and ensuring civil exchanges is crucial to facilitate learning.

MOOCs offer a variety of communication platforms such as peer grading for feedback, automated feedback for quizzes, social networking, and asynchronous discussions [13]. Commonly used in almost all online courses, asynchronous discussion forums are a staple of the MOOC delivery system. Often unstructured, unsupervised, and with optional participation, a MOOC discussion forum is considered to be a means of developing a peer-supported learning environment [12]. In addition, discussion forums may contain some of the richest data because they allow students to engage in collaboration and cocreation of knowledge resources to reach mutual goals [12]. Furthermore, the asynchronicity of forum discussions gives students time to reflect and process information before they add their input [14]. However, the relative anonymity of discussion forums also seems to encourage less civil behaviors that could be destructive [15].

### Objectives

This study aimed to examine the environment created by a medicine and health care MOOC by studying students’ posts in the course’s Web-based discussion forum. The objectives were to (1) examine the prevalence of posts deemed disrespectful, insensitive or disruptive, and inconducive to learning; (2) describe the patterns and types of uncivil posts; and (3) highlight aspects that could be useful for MOOC designers and educators to build a culture of cybercivility in the MOOC environment. The findings of this study will inform the parameters of future investigations and suggest preventive measures to deal with cyberincivility in the MOOC learning environment.

## Methods

### Context of the Study

The data used in this research came from the discussion forum of the MOOC *Medical Neuroscience*, offered by a large private university in the southeast region of the United States. Designed for first-year students in graduate-level health professions programs, this course was taught by a long-time professor in the university’s physical therapy department [16]. Initially launched in 2014, this course was offered 4 times. Each time, students were required to take the course as a cohort, working at the same pace. In 2016, the course relaunched as an on-demand course in which students could take the course at their own pace. Students were loosely grouped into cohorts based on enrollment dates, but those who wanted to take more time to complete the course were automatically rolled over to the next cohort.

The sociodemographics of the learners enrolled in the course diverge in a few ways from the typical MOOC enrollment profile, and most of the university’s MOOCs as well [17]. First, 50% of learners in the Medical Neuroscience MOOC were females; Bayeck’s review of the literature found that that the number was typically around 43% [17]. Second, a much higher-than-average percentage of learners enrolled in the Medical Neuroscience MOOC were current students somewhere at the time of enrollment. Reportedly, the average of full-time students across all Coursera courses is 28% [17]. In this course, 41% of learners were full-time students, and another 11% were attending part-time. As a result of the course enrolling a higher percentage of current students, a lower-than-average percentage of participants already had a college degree (73% vs 77% Coursera average) or were employed (65% vs 74% Coursera average) [17]. Consistent with courses across the Coursera platform, 25% of learners were located in the United States. Because our sociodemographic data are based on a survey conducted by Coursera in 2014, individuals who joined Coursera after 2014 are not reflected in the data. While we have no evidence of a marked shift in the sociodemographics since then, there may be some differences in the numbers we report.

### Data Collection

The data were collected on May 9, 2017. At that time, the course had already been available on demand for 11 months and had enrolled approximately 56,000 students. About 25% of these students subsequently became *active learners* in the course. Active learners are defined as students who enroll in a course and watch at least one video, attempt at least one assessment, or participate in the discussion forum as either author or viewer [18]. The course included a discussion forum where students could post information/questions, known as initiating posts, and respond to posts from other learners.

One of the biggest challenges associated with analyzing discussion forum data is that the data files contain a lot of “noise”—records that do not contain valid, user-entered data. In the files obtained for this analysis, out of 21,101 posts in the dataset, 12,396 (58.75%) were cleaned from the final research data file because they contained one or more types of invalid data. While this initially appears to be a high percent of missing data, it is typical for our Coursera courses given some of the technical difficulties inherent in how these data are recorded and retrieved. This is primarily attributed to the way data are stored in the structured query language files on the back-end of the course platform, in that most of these unusable data records never contained valid data. For example, if a learner clicks on a button to generate a reply to a discussion post and then cancels that action, a record remains in the dataset, indicating the initial attempt, but there are no valid data in the said record. The data file we obtained for this analysis records such events with empty HTML tags such as “<co-content><text></text></co-content>.” We determined that these records were not valid data and, therefore, should be cleaned up without analysis. Other learner actions that generate data records, which are not analyzable, include creating a post that contains only symbols, images, emojis, or external links.

We identified 2 types of posts that potentially included valid data, but that we removed from our analysis. The first of these were duplicate posts that likely occurred owing to a technical problem on the user end. For example, the dataset contained 12 posts that all read, “I consider myself to be a lifelong learner and decided to take this course because it seemed interesting and challenging. Good luck to everyone!”; these were all posted within a span of about 10 seconds. In cases such as these, we retained only the first post and removed the duplicates from our dataset. Finally, we determined that some data records were truncated or recorded as a string of characters at the point that certain HTML codes were entered manually by a learner. If that happened at the beginning of a post, the subsequent data were lost. An example of this is a post that was recorded as, “<co-content><text>ÿ¨ÿ≤ÿßŸÉ ÿßŸÑŸÑŸá ÿÆŸäÿ±ÿß</text> </co-content>.” Because we have no way to reconstruct what this post originally said, we deleted it from the dataset.

**Figure 1:**
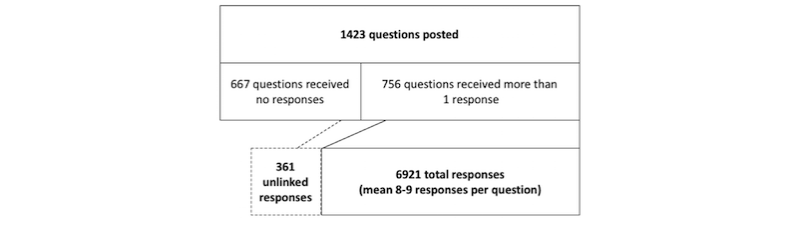
Data file record types.

Thus, the final dataset in our analysis included 41.25% (8705/21,101) posts consisting of the following: (1) 667 questions that received no responses; (2) 756 questions that received at least one answer; (3) 6921 responses that applied to 756 posts; and (4) 361 responses where the initiating post was unknown (Figure 1).

### Data Analysis and Rigor

Data management and analysis were performed with NVivo 12 (QSR International Pty Ltd.). The unit of analysis was each post in a MOOC discussion thread. For analysis, posts were organized by question title, question text, and answer text. A comment in the forum may contain any combination of question title, question text, and answer text. In other words, if a comment included a question title and question text, it was coded and counted as 2 posts.

**Figure 2:**
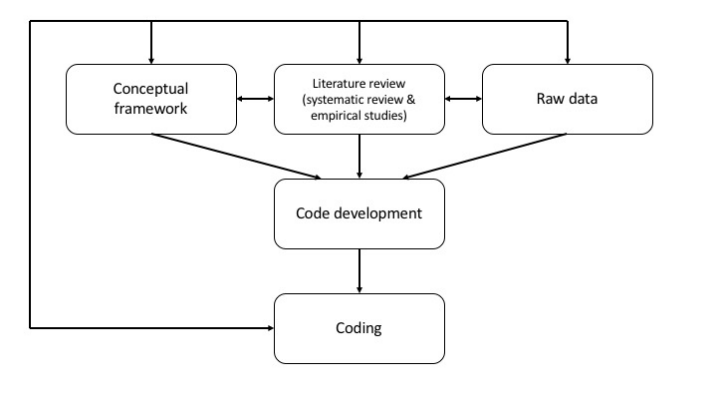
Iterative process of coding.

Uncivil Web-based behavior is that which does not conform to norms or values held by most members of society [19]. In analyzing 8705 posts, we considered uncivil posts as “features of discussion that convey an unnecessarily disrespectful tone toward the discussion forum, its participants, or its topics” [20]. Our definition is consistent with the definition of cyberincivility set forth by De Gagne et al [1]. Putting this definition in the context of MOOC learning, the coding team sorted out uncivil posts first. When gray areas existed, the coding team members asked themselves whether they would have posted such a comment (considering both content and communication style) in the discussion forum; if their answer was no, then the comments were coded as uncivil. Using the iterative process of coding, a series of *a priori* codes were developed from the conceptual framework [20-22], the systematic review [1], and the empirical studies of cyberincivility [23,24] (Figure 2).

Codebook: A list of codes and their definitions as needed (a priori codes (1-25); emergent codes (26-27); *a priori codes not found in this study).

1. Ambiguous or vague responses (a lack of clarity in meaning, imprecise, or unclear use of language)
2. Becoming offended easily by opposing ideas posted on Web (being unnecessarily critical or unfriendly toward others)*
3. Blaming technology for failure of communication, assignment completion, or submissions
4. Breaching patient’s privacy*
5. Challenging faculty knowledge or credibility
6. Cheating on exams or quizzes
7. Criticizing course or instructor publicly
8. Criticizing non-traditional sub-cultures (negativism toward groups other than one's own)
9. Derogatory remarks about another profession*
10. Derogatory remarks about one’s institution*
11. Does not contribute to the conversation (lacking responsiveness and engagement)
12. Does not relate to content (off topic)
13. Failing to complete assignments in a timely manner
14. Failing to fulfill group responsibilities
15. Flooding a Web-based environment with comments or messages
16. Making personal attacks or threatening comments*
17. Making racial, ethnic, sexual, or religious slurs*
18. Posting others’ personal information*
19. Posting short, terse responses (abrupt posts that do not add meaning to the discussion)
20. Refusing to participate in required Web-based discussions
21. Spelling or grammar errors, incomplete sentences
22. Taking credit for others’ work (not giving proper credit for someone else’s ideas)*
23. Too casual (use of smiley faces or linguistic shortcuts, joking, colloquial, too personal)
24. Using displays of attitude such as capitalizing or boldfacing
25. Vulgarity (use of cursing, swearing, or profane words or foul languages or expressions)
26. Posting in a non-English language
27. Making a provocative statement (remarks that trigger emotional reactions)

In addition, each coder’s reflective, analytic memos enhanced interpretations of the findings [25]. Most codes were self-explanatory, while others needed to be defined in the context of the study. The team collaboratively developed a set of short definitions for each code to ensure for clarity and consistency in the analysis (Textbox 1). Among the codes presented in the textbox, 1-25 are *a priori* codes (of which 2, 4, 9, 10, 16-18, and 22 were not found in this study) and 26-27 are emergent codes.

Coders (BEH, HKP, and JCDG) had regular meetings and cross-checked the codes to ensure a high degree of reliability [26]. They coded the posts to uncivil posts based on the *a priori* codes, then compared their results, and discussed disagreements, as well as emergent codes. Using the consensus approach, the team agreed to all codes applied to all posts flagged as uncivil [27].

After coding each post, the team collaboratively structured all codes into themes. Clark’s conceptualization of the *continuum of incivility* guided the development of 3 broader themes— annoyance, disruption, and aggression—depending on the degree and impact of the uncivil posts [28]. Annoying posts were defined as those that did not interrupt the teaching and learning process but may have had an impact on the learning environment [22]. Disruptive posts were those that substantially or repeatedly impeded either the instructor’s ability to teach or the students’ ability to learn [29]. Aggressive posts were defined as those amounting to intimidation, humiliation, violence, or breach of confidentiality, all of which being likely to bring emotional pressure on members of a teaching and learning community [22]. Posts that contained >1 kind of *a priori* codes were assigned multiple codes.

## Results

### Prevalence of Uncivil Postings

**Figure 3:**
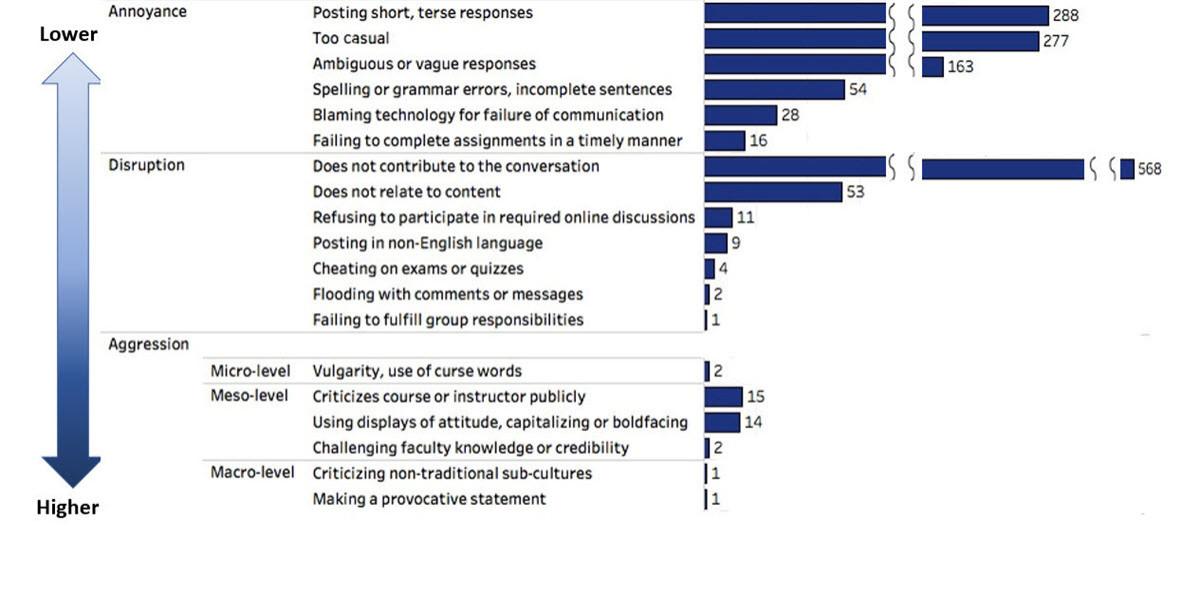
Themes and frequencies of uncivil postings.

Of 8705 posts included in the analysis, 1043 (11.98%) were considered as the presence of uncivil posts and 7333 (84.24%) as the absence of uncivil posts, while 329 (3.78%) were treated as missing data as they were uncodable (ie, HTML code, random characters, repeated entries that indicate a data processing error). As shown in Textbox 1, of 25 *a priori* codes, 8 were not present in this study, and 2 new codes emerged (ie, posting in a non-English language and making a provocative statement). These 19 *a priori* codes were organized under the themes of *annoyance*, *disruption*, and *aggression*. Of 1043 uncivil posts, 466 were coded into >1 *a priori* codes, which rendered 1509 instances. Of those 1509 total instances, 826 (54.74%) were put into annoyance, 648 (42.94%) into disruption, and 35 (2.32%) into aggression. Figure 3 depicts the occurrences of each code in each theme.

#### Annoyance

Of 826 instances that were in the “annoyance” theme, short or terse responses were most common, followed by too casual (eg, “jajaja, i agree with you.”). About one-fifth of posts contained ambiguous or vague responses (eg, “why we call the eyes the window of soul. It is because retina derived from brain [diencephalon], so our thought process reflected in eyes, either we say true or false”). Less common were posts that contained spelling or grammar errors, blamed technology for a miscommunication, and failed to submit or complete an assignment in a timely manner.

#### Disruption

Of 648 instances in the “disruption” theme, posts that did not contribute to the discussion were most common. In addition, students posted comments that were not related to the course content. For instance, one post declared, “I just stop in front of the amazing brain which God give us and I can say how much is the mighty of God!!!” Students also refused to participate in the discussion by posting, for example, “sorry, I’m not into this kind of task.” Some openly disclosed acts of academic dishonesty. For example, “I only skimmed through the first week and took the quiz without watching all the videos. Is there any way to mark the videos as watched without watching them?” Posts that flooded the discussion forum with self-bragging or complaints and failed to fulfill group responsibilities or group assignments were also found.

#### Aggression

A total of 35 instances in the “aggression” theme were divided into 3 scales of aggression based on the scope of impact—(1) microaggression (individuals or group members); (2) mesoaggression (the learning community as a whole); and (3) macroaggression (societal or global effect outside of the learning environment). First, as shown in Figure 3, microaggressive comments were present but uncommon (eg, “Damn, this is a lot of information to learn in one week...someone got any tips?”). Second, mesoaggressive comments were found more frequently. The most common type of mesoaggression was a criticism of the course or instructor (eg, “I will complete this course of study for merit. There are high school students that are studying Neuroscience at an equivalent level. Disappointed.”), followed by displaying aggressiveness by capitalizing or boldfacing. In addition, challenging of faculty knowledge or credibility was noted.

**Figure 4:**
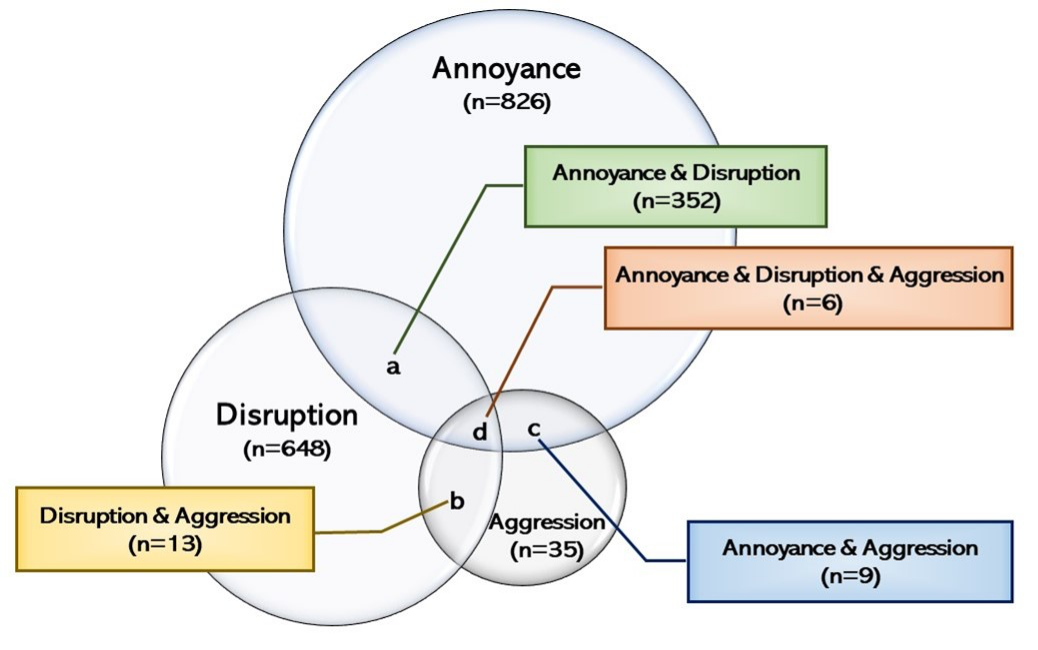
Overlaps between annoyance, disruption, and aggression.

Finally, 2 posts revealed macroaggression. One post criticized nontraditional subcultures as in “My apologies to our Chinese colleagues—I realize my above comment just sounded horrible.” The other made a provocative statement in regard to a discussion thread, saying “a person is no longer a person, if they become disabled (or belong to a group persecuted in Nazi Germany)... The Nazi’s believed that homosexuals, mentally retarded, deformed individuals (ugly), gypsies, Jews and others were non-persons...the Nazi’s systematically murdered (exterminated) those persons.” While this post was confusing and it was difficult to discern the intention of posting, the coding team considered it a substantial risk to conflict within and outside the learning environment.

### Patterns of Uncivil Posting

While some posts were coded to have only one type of *a priori* code, 466 posts had >1 code. Of those 466, 380 posts were attributed to 2 or 3 themes. Within those 380, 352 (92.6%) overlapped both “annoyance” and “disruption,” 13 (3.4%) overlapped both “disruption” and “aggression,” 9 (2.4%) overlapped both “annoyance” and “aggression,” while 6 (1.6%) intersected all 3 themes (Figure 4).

#### Annoyance and Disruption (n=352)

The heaviest overlap was between the disruption and annoyance groups (n=344). A further breakdown of posts “not contributing to the conversation” revealed that most posts were too casual (n=232), ambiguous or vague (n=42), contained spelling or grammar errors, or incomplete sentences (n=25), and blamed technology for a communication failure (n=24). For example, this post was coded as not contributing to the conversation (disruption) and too casual (annoyance): “I meant to type idea...not Ida. Ugh, the autocorrect feature on my iPad touch keyboard is killing me! Lol.” A few posts considered “too casual” also signaled a refusal to participate in required Web-based discussions (n=7). Examples of these posts were “Not now, thanks!” or “SORRY, NO TIME.”

#### Disruption and Aggression (n=13)

Most disruptive uncivil behaviors in the discussion forum overlapped with mesoaggression (n=12). Within mesoaggression, posts criticizing the course or instructor publicly and posts using displays of attitude were the most frequent. For instance, the most common intersection of coding was between posts criticizing the course or instructor publicly and posts not contributing to the conversation (n=4). One such post asked, “How can I follow if the lecturer is online or offline? or does the lectures occur in a way of offline that there is no active online classes? someone make me to understand that point plz...” Four posts were coded to displays of attitude (mesoaggression), as well as not contributing to the conversation (disruption), such as “FASTER” or “ITS GOOD LECTURE.” Within macroaggression, one post was coded as both not contributing to conversation and making a provocative statement. One post was coded to disruption and macroaggression, and no posts were coded to disruption and microaggression.

#### Annoyance and Aggression (n=9)

Uncivil posts that fell into the annoyance group overlapped with the meso level of aggression; nothing was coded to annoyance and microaggression or macroaggression. Within mesoaggression and annoyance, 7 posts criticized the course or instructor publicly and were considered annoying. One example, “IT IS POSSIBLE TO EXTEND THE TIME FOR THE COURSE OF THIS FIRST WEEK BECAUSE FOR ME IT IS A LITTLE MORE SLOW TRANSLATION AND THIS SO INTERESTING THAT I SHOULD GO TAKE NOTE I WOULD APPRECIATE YOUR HELP,” was coded as failing to complete assignments in a timely manner (annoyance) and using displays of attitude, capitalizing, or boldfacing (mesoaggression).

#### Annoyance, Disruption, and Aggression (n=6)

Six posts were coded as all 3 types of uncivil behavior. For example, a post stating that the course was “too hard” was coded as too casual (annoyance), short, terse responses (annoyance), does not contribute to the conversation (disruption), and criticizes course or instructor publicly (mesoaggression). Another post, “I didnt [sic] realise I should delete my name and retype it. Now I miss my deadline. I feel like quitting. Not fair!” was coded as blaming technology (annoyance), does not contribute to the conversation (disruption), and criticize course or instructor publicly (mesoaggression).

## Discussion

### Principal Findings

Using the data from Web-based discussion forums, this study investigated the prevalence, content, and characteristics of uncivil posts made by students in the MOOC Medical Neuroscience. Results indicate that the majority of posts in our sample contained no uncivil behavior. Those that did tended to be annoying and disruptive rather than aggressive. This finding suggests that, overall, students participating in the MOOC used the discussion forum appropriately to engage in the material with others. With that said, the most prevalent form of uncivil posts was “does not contribute to the conversation” (568/1509, 37.64%), suggesting that students did not always use the discussion forum effectively for learning purposes.

These findings are similar to those of other studies. Bonafini et al analyzed forum posts in a Creativity, Innovation, and Change MOOC and found learners’ posts to be mostly “polite and friendly” [30]. While the researchers did not analyze the relevancy of posts, they did analyze the level of learning and determined that the posts indicated students were learning about each other and the content, but not demonstrating deep learning or critical thinking. Within a closed Web-based forum experiment, Berg compared posts on a controversial topic with a noncontroversial topic in both an anonymous and nonanonymous format; the researcher found no posts coded for incivility (defined as verbalizing threats or assigning stereotypes) and a more respectful tone present in the forum around the controversial topic regardless of anonymity [31]. In addition, irrelevant posts were common at 14% for the controversial topic and 8% for the noncontroversial, suggesting that topic had a larger impact on the discussion quality while anonymity had no effects [31]. A future study may be warranted to explore how controversial topics would affect the quality of discussion forum in a MOOC environment.

Our study did not reveal 8 *a priori* codes that were widely addressed in cyberspace (see Textbox 1); this could be attributed to a number of factors. Foremost, it is likely that our population of premed students were not yet working with other medical personnel to care for patients. Therefore, they had no ability to breach a patient’s privacy, such as by sharing their real-world experiences. In addition, they would have had no remarks—derogatory or not—to share regarding another professional or their own institution. The anonymity of the MOOC environment itself, perhaps, led to the absence of posting others’ personal information and taking credit for others’ work, which may be easier to do in an environment where learners know each other. The absence of several codes, namely becoming offended by opposing ideas, making personal attacks, and making racial, ethnic, sexual, or religious slurs may relate to the content of the MOOC itself. For example, Coe et al found that papers centered on health topics garnered a lot fewer uncivil posts in a Web-based news forum than did topics such as sports, politics, economics, crime, and taxes [20]. The characteristics of the population and the nature of the subject might explain why certain uncivil behaviors found in Web-based discussions were absent in our study findings.

The 2 emergent codes in this study occurred infrequently and relate to 2 separate phenomena. The first—posting in non-English language (n=9, 0.6%)—was considered a disruption to the learning environment where the use of English was expected in Web-based forums. Considering that 75% of learners in this study were located outside the United States, its infrequency is somewhat surprising. However, other studies show that nonnative English-speaking students forgo posting in Web-based forums when they lack confidence in their English writing skills [32], when their cultures do not place a high value on dialogue [33], and when their learning preferences do not include group discussions [32]. Therefore, it could be that nonnative speakers opted not to post questions, thereby reducing the instances of this emergent code. Likewise, some international students, including Koreans, may learn English with a focus on correct grammar rather than on speaking and listening skills; these students may decide not to post to forums [32]. In this study, there were 3.58% (54/1509) of spelling and grammar errors that could be interpreted as an annoyance to other learners; these instances may prove to be a minor price for the much larger added benefit of encountering diverse perspectives from a variety of countries.

The second emergent code, making a provocative statement, occurred only once but is noted to highlight the potential harm of this form of incivility. We defined provocative statements as those that trigger emotional reactions. Gervais considered similar statements when conducting an experiment to study incivility in an Web-based forum with a political topic [34]; in this study, participants were subjected to uncivil posts, including extreme statements and hyperbolic spins defined as “use of an inflammatory word or phrase that makes individual or action seem more radical, immoral, or corrupt,” as well as histrionics, which included language suggesting an “individual or group should be feared or is responsible for sadness” and the inclusion of emotional cues like the use of exclamation points and uppercase letters [34]. Histrionics, especially the added exclamation points and capitalization, heightened feelings of anger, and offensiveness led to more uncivil reactions. Although this study was specific to political discourse, it shows the importance of paying close attention to this type of behavior within MOOC discussion forums.

The Web-based discussion forum is one of many ways for students to engage in the learning material within MOOCs. Studies indicate that students differ in their levels of participation in forums, assessments, and lecture content [35], and those who participate in discussion forums tend to have higher completion rates [30,35-37]. At the same time, students identify discussion forums as a source of frustration for their potential to contain rude posts and cause information overload owing to their sheer volume [38]. In response, MOOC developers and instructors would do well to maintain a civil discourse within discussion forums and decrease off-topic and redundant posts and discussion threads.

To minimize irrelevant posts and decrease the volume of learner-created threads, instructors could prepopulate forums with threads related to specific weekly content or themes [38]. In addition, instructors could clearly label threads meant to answer students’ course-related questions from those meant to engage other students in topical discussions. This way, instructors can intentionally create discussion prompts that would lead to conversations consisting of higher levels of learning—from critical thinking to applying course concepts [30].

MOOC instructors can increase their presence by interacting with students in Web-based forums. Effective ways of doing so include beginning with a greeting, using learners’ names, and incorporating self-disclosure of one’s own real-world experiences, opinions, and values [39]. In addition, instructors can hire and train teaching assistants to monitor discussion forums, to answer students’ questions promptly, and to steer conversation as needed [40]. MOOC instructors might best support a culturally inclusive learning environment with additional visual and audio aids [41], translating content into one or more languages [40], and facilitating multicultural learning communities within Web-based discussion forums [42]. Scheduling live video-streamed discussions would provide students—native and nonnative speakers alike—another opportunity to engage with the course content and the instructors in a different format [38]. To further support students, these live discussions could be held at varying times throughout the week and recorded for asynchronous viewing [43].

### Limitations

This study has several limitations. First, our findings represent a single MOOC enrolling learners interested in neuroscience. Therefore, our analysis may not be representative of other MOOCs, including those focused on the humanities, social sciences, and other subject disciplines. Second, our analysis of posts lacked context; in other words, we were unable to read posts in the order in which they were posted to determine how an uncivil post affected later posts. Third, our data were deidentified, and as anyone can enroll in a MOOC, we could not discern if a particular post represented a single person or possibly multiple individuals enrolling in the MOOC and working together. Finally, it is difficult to standardize personal opinions about what interactions are considered uncivil in Web-based communication—especially in a MOOC environment.

### Future Directions

Further studies might compare our findings to those in other MOOCs. It could be that certain courses, such as one focused on politics or current events, would garner more instances of incivility. One study, for example, found a higher prevalence of incivility (22%) in Web-based discussions on the Arizona Daily Star Web-based news site compared with the prevalence of incivility we found (12.0%) [21]. It would be worth determining if the topic area or the platform had more to do with the prevalence of incivility. Another study found a much lower prevalence of incivility (4.6%) when analyzing 8934 tweets from nurses and nursing students [44]. Nurses were involved in uncivil behavior that included profanity, product promotion that lacked evidence, and both interprofessional and intraprofessional aggression. Therefore, it would also be worthwhile to study a MOOC geared more toward health professionals and others working in the field to determine whether the prevalence and types of incivility would shift from those found in our study.

### Conclusions

To the best of our knowledge, this is the first study to explore the phenomena of cyberincivility in the health-related MOOC toward the education of future health care professionals. In the current age of interconnectivity and the internet, cyberincivility is a challenging concept as it is difficult to create a set of universal standards for what we as educators and students consider civil or uncivil cyber behavior. However, there are certainly gross examples of cyberincivility in almost all forms of Web-based communication. Many of these issues arise during every day in-person communication as well, but the lack of face-to-face interaction on Web exacerbates the problem. In addition, owing to the worldwide reach of MOOCs, differences in culture and language often lead to misinterpretations. While accessibility and affordability add to the attractiveness of MOOCs in health professions education, the relative anonymity of this environment may encourage bolder and less civil discussions than those occurring in closed online courses. Our findings contribute to the body of knowledge into a deeper understanding of cyberincivility in Web-based learning; these also offer some insights useful both to MOOC designers and educators in enhancing student learning. It would be worthwhile to conduct more empirical research that explores issues around cyberincivility, their possible impacts, and the implications for MOOC practitioners. This type of work may help education policy makers to understand better how to create a culture of cybercivility in the MOOC environment.

## Abbreviations

MOOC: massive open online course
